# Urachal Sinus Complicated by an Umbilical Abscess

**DOI:** 10.7759/cureus.9527

**Published:** 2020-08-02

**Authors:** Talal Almas, Muhammad Kashif Khan, Mishal Fatima, Faisal Nadeem, Muhammad Faisal Murad

**Affiliations:** 1 Internal Medicine, Royal College of Surgeons in Ireland, Dublin, IRL; 2 Surgical Oncology, Federal Government Poly Clinic (Post Graduate Medical Institute), Islamabad, PAK; 3 Surgical Oncology, Maroof International Hospital, Islamabad, PAK; 4 Obstetrics and Gynecology, Rawalpindi Medical University, Rawalpindi, PAK; 5 General Surgery, Maroof International Hospital, Islamabad, PAK

**Keywords:** urachus, sinus, abscess, umbilical

## Abstract

The urachal sinus is a vestigial remnant that ensues in the aftermath of incomplete obliteration of the embryonic urachus. Urachal sinuses often remain asymptomatic, being discovered incidentally in instances where they are complicated with a superimposed infection or abscess. Due to their rare occurrence in adults, urachal sinuses are rarely included in the list of differential diagnosis surrounding umbilical pain in adult patients. We hereby delineate a unique case of a urachal sinus in a 26-year-old male patient. Due to the presence of an abscess in a hirsute male, a presumptive diagnosis of an umbilical pilonidal sinus was suspected. However, further diagnostic workup divulged an unequivocal diagnosis of a urachal sinus complicated by an abscess formation.

## Introduction

The urachus is a vestigial tubular structure that connects the umbilicus with the urinary bladder during the fetal life and subsequently involutes after fibrosis [1]. In rare instances, it may persist as a urachal sinus and thus present with vague lower abdominal pain or umbilical discharge [2]. Such urachal anomalies most commonly afflict the pediatric population; however, on exceedingly rare occasions, urachal sinuses can also present in the adult population [2,3]. When they do present in adults, urachal sinuses can masquerade as a plethora of umbilical pathologies, such as a persistent omphalomesenteric duct and omphalitis [4]. This notion renders their apt diagnosis a medical conundrum [4]. We hereby delineate a rare case of an adult hirsute male who presented with an infected urachal sinus. His clinical manifestations and physical abdominal examination insinuated a diagnosis of an umbilical pilonidal abscess, which routinely presents in hirsute males. However, further diagnostic investigations revealed the presence of an infected urachal sinus.

## Case presentation

**Figure 1:**
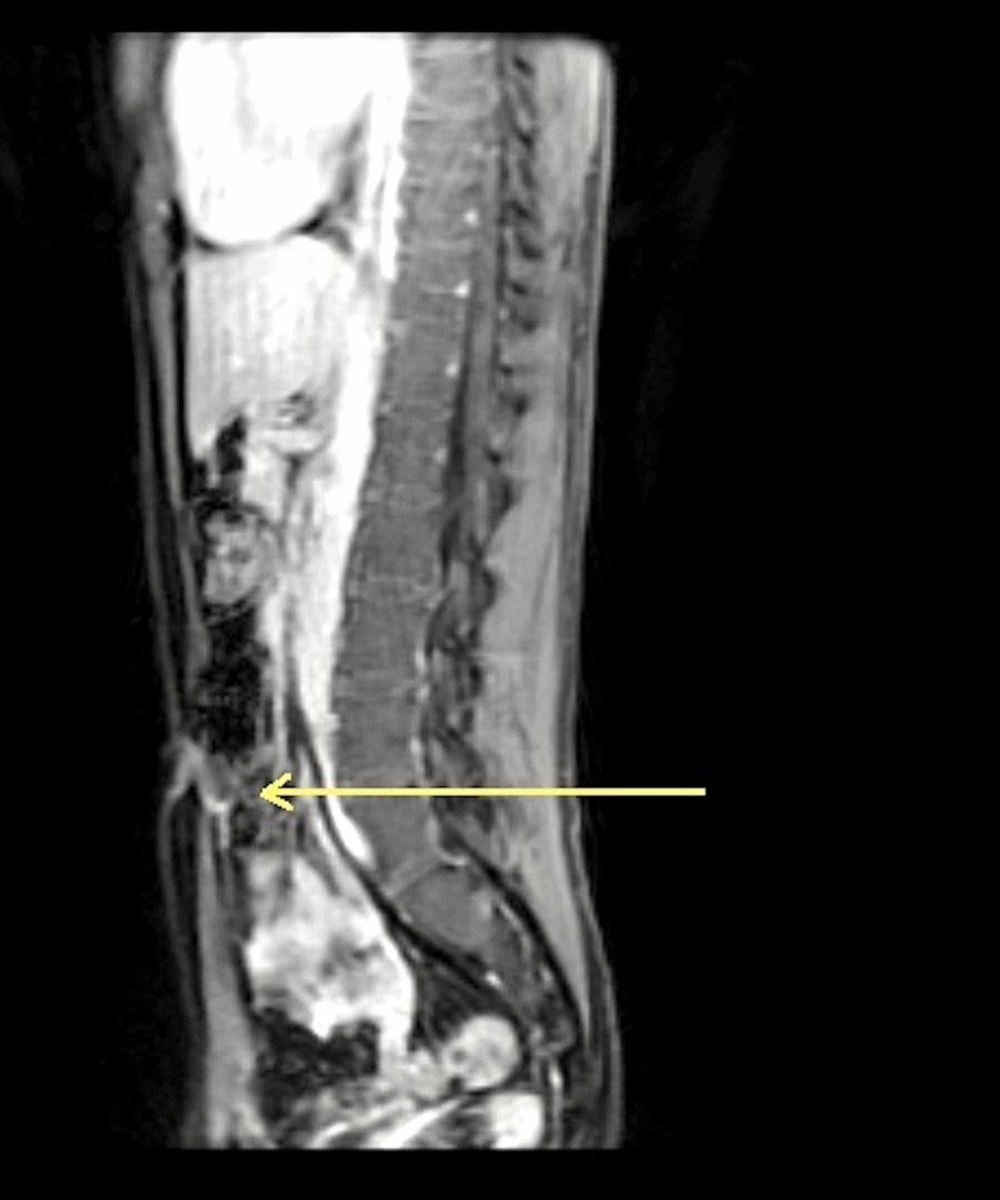
An MRI scan of the abdomen and pelvis delineating a urachal sinus (arrow) complicated by abscess formation.

We elucidate an interesting case of a 26-year-old male with no comorbidities who presented to our department with a complaint of purulent discharge from the umbilicus. The purulent umbilical discharge was accompanied by intermittent umbilical pain for the past month. Pertinently, the patient also reported a history of burning micturition of recent onset. Physical examination of the umbilicus, along with the abdomen, revealed a hirsute male with a deep, erythematous umbilicus with a purulent discharge. Coupled together, the physical examination and the patient’s presenting history alluded towards a presumptive diagnosis of an umbilical pilonidal sinus (UPS), which commonly presents in a hirsute male with a deep umbilicus. However, owing to recent urinary complaints, a urachal communication was suspected. A subsequent urinalysis was unremarkable. To ascertain the underlying pathology, an MRI scan of the abdomen and pelvis was conducted. MRI was considered the imaging modality of choice because MRI, in addition to elucidating the underlying urachal anomaly, also shows the extent of the infection and the potential involvement of the adjacent organs. The MRI scan divulged a mild peripheral wall enhancement below the umbilicus. Pertinently, there was no communication with the bladder observed. Furthermore, the MRI scan revealed a fluid-containing cystic lesion measuring 17 x 18 x 23 mm, insinuating a diagnosis of a urachal sinus (Figure 1). 

Subsequent evaluation by the consultant radiologist further reaffirmed the presence of a urachal sinus complicated by an abscess formation. Thereafter, the patient was duly informed of the change in scenario and the final diagnosis. The patient was subsequently admitted and started on broad-spectrum antibiotics. A culture of the purulent exudate did not grow any organisms, likely due to the prompt commencement of antibiotics. An omphalectomy along with excision of the entire tract was performed under general anesthesia. A histological analysis was performed and was unremarkable for any malignant transformation. Postoperatively, the patient was discharged within 24 hours. On the fifth day postoperatively, the patient presented with mild seroma formation (15 mL) at the wound site; aspiration of the seroma was thus performed, and the seroma subsequently abated within the three following days. The patient continues to thrive without any additional complications. 

## Discussion

Pathologies evoking the involvement of a urachus remain exceedingly rare. Persistence of a urachal sinus is a rare clinicopathological entity that boasts a meagre prevalence of one in 5,000 live births and usually presents in male children [5]. Its occurrence in adults is even rarer, with a prevalence rate hovering around 2% of the total reported cases [6]. Since urachal sinuses are predominantly encountered in the pediatric population, they often do not merit a place in the list of differential diagnosis surrounding umbilical discharge or lower abdominal pain in an adult male. 

A perusal and evaluation of the published medical literature reveals that cases of infected urachal sinuses can manifest with a vague constellation of clinical symptoms, including umbilical discharge, erythema, lower abdominal pain, fever, and burning micturition [6,7]. In a multitude of instances, a urachal sinus can be secondarily infected, usually with Escherichia coli, and form an abscess, thereby leading to abdominal pain and a purulent umbilical discharge [8]. Pertinently, our patient presented with a clinically similar picture. However, in our case, the patient was a hirsute male with a deep umbilicus, factors that are established risk factors for the development of a UPS [8]. Due to the presence of these factors, the clinical picture alluded to a diagnosis of a UPS. Nevertheless, further diagnostic evaluation using MRI divulged the unequivocal presence of an infected urachal sinus, which was thus established as our final diagnosis. 

A multitude of studies purport the notion that multimodal imaging techniques remain pivotal in aptly construing a diagnosis of an infected urachal sinus in the adult population [4,7]. Although they are seldom encountered in adults, urachal sinuses ought to be considered in the list of differential diagnosis pertaining to adult male patients presenting with lower abdominal pain and intermittent umbilical discharge. The differential diagnosis, in such instances, usually consists of pathologies such as a UPS and omphalitis. While prompt treatment can effectively resolve the pathology, a dilatory diagnosis can result in adverse outcomes such as the development of fistulas and systemic sepsis [2,7].

In our case, considering the eventual diagnosis of a urachal sinus, an omphalectomy with complete resection of the urachal remnant and the entire remnant tract was deemed apt. Omphalectomy therefore remains the treatment of choice since conservative incision and drainage of the abscess may lead to recurrence of the abscess in the tract. Furthermore, the tract can serve as a nidus for future infections. In accordance with this notion, surgical literature vouches for surgical excision as the preferred treatment modality [6,7,9]. Appropriate guidelines accentuating the optimal management of infected urachal sinuses to ameliorate the associated therapeutic outcomes are therefore warranted.

## Conclusions

Infected urachal sinuses are an exceedingly rare clinicopathological entity. Their presence in hirsute adult males can often mimic umbilical pilonidal abscesses, obscuring a timely diagnosis. Imaging modalities, such as MRI, play a pivotal role in yielding a timely and accurate diagnosis. Due to the risk of future infections, complete surgical resection of the entire tract remains the preferred treatment modality.
